# Photochemical Reactions in Dialdehyde Starch

**DOI:** 10.3390/molecules23123358

**Published:** 2018-12-18

**Authors:** Marta Ziegler-Borowska, Katarzyna Wegrzynowska-Drzymalska, Dorota Chelminiak-Dudkiewicz, Jolanta Kowalonek, Halina Kaczmarek

**Affiliations:** Faculty of Chemistry, Nicolaus Copernicus University in Torun, Gagarina 7, 87-100 Torun, Poland; kasiawd@doktorant.umk.pl (K.W.-D.); dorotachelminiak@wp.pl (D.C.-D.); jolak@umk.pl (J.K.); halina@umk.pl (H.K.)

**Keywords:** starch modification, dialdehyde starch (DAS), cross-linkers, UV-irradiation, surface properties

## Abstract

In this study potato and corn starch were subjected to oxidation, using sodium periodate, to obtain dialdehyde starch (DAS) containing different amount of aldehyde groups. The obtained modified starch samples have been characterized with chemical analysis, scanning electron microscopy (SEM) and ATR-FTIR spectroscopy. Then, the samples were exposed to polychromatic UV radiation and the course of photochemical reaction has been monitored with ATR-FTIR spectroscopy. The surface properties of the native and dialdehyde starch before and after UV-irradiation have been determined by contact angle measurements and calculation of surface free energy. The crystallinity of the samples has been estimated with X-ray diffraction (XRD). It has been proved that the dialdehyded corn starch contained a higher amount of functional groups was more photostable than the oxidized potato starch. Sodium iodide(V), firmly bound to DAS macromolecules, has been found to have a significant effect on the photooxidative degradation of the tested systems. In addition, the mechanism of photoinduced reactions in the dialdehyde starch has been proposed.

## 1. Introduction

Polysaccharides are of great interest to scientists owing to the ease of modifications, which raises the possibility of their use in numerous new sectors of the industry [1,2,3,4,5]. The unquestionable advantage of the materials based on polysaccharides is that the raw materials come from renewable sources, which is consistent with current environmental trends. Moreover, the large availability of starch and its low price are further benefits. Moreover, starch is the component of biodegradable plastics, which makes the products with starch and its derivatives environmentally friendly materials. One of the widest applications of starch and dialdehyde starch due to their biocompatibility is the use of these polysaccharides in the synthesis of biomaterials and biodegradable plastics [6,7,8].

Oxidation of starch with efficient oxidizing agent, such as periodate, i.e., metaiodate (VII), is the way to change significantly the properties of native starch. Periodate oxidation is an extremely specific reaction to transform vicinal di-hydroxyl (glycol) groups to paired aldehyde groups without significant side products. The selectively oxidized polysaccharide contains two aldehyde groups in each repeating unit of macromolecule. These aldehyde groups are formed by the opening of the glycosidic ring as a result of preferential C2–C3 bond cleavage [1,9,10].

In the middle of the last century, the oxidation reaction became the industrial method of obtaining dialdehyde starch [11,12]. The conversion rate of this process is generally very high (80–90%). In this technology, periodate, i.e., iodate (VII) (IO_4_^−^), is reduced to iodate (V) (IO_3_^−^) and can be re-used after oxidation.

Such modified starch is insoluble in cold water but soluble in hot water, however, its solubility (dependent on oxidation degree) in organic solvents is changed. DAS does not form characteristic complexes with iodine. The aldehyde groups in DAS are present as hemiacetals or hemialdals rather than free aldehydes.

A further modification of starch may lead to a cross-linked system, which results in a complete lack of solubility and simultaneously in improvement of mechanical properties [13,14]. The ability of DAS to crosslink is used in production of adhesives, plastics, leather, textile, and paper (where it is a wet-strength additive).

The presence of highly reactive aldehyde groups in the oxidized starch is desired for potential medical and biomedical applications. In fact, application of the oxidized starch in tissue engineering (as a material improving the protein adsorption) and as a drug delivery carrier has been described [15]. Recently, it was also reported that DAS has biocidal properties against bacteria and viruses [16,17,18].

Although a lot of research was dedicated to the structure and characteristics of the physicochemical properties of DAS [9,10,11,12,13,14,15,16,17,18], but there is no information on its photostability. Due to the fact that both starch and dialdehyde starch are widely used to obtain biomaterials and bioplastic, testing its photostability depending on the source is important. Polychromatic UV radiation is commonly used primarily for the sterilization of materials; hence the study of its impact on the starch and dialdehyde starch provides important information on the functional properties of starch-based materials. The purpose of our work was to study the influence of polychromatic UV radiation on dialdehyde starches with different content of functional groups. The modified starches were obtained by chemical oxidation of starch from two different plant sources: potato and corn.

Moreover, the second objective was to estimate the ability to modify DAS surface by UV radiation. Thus, special attention was paid to the study of the surface properties of the films based on the oxidized starches which are very important for biomedical applications. It should be pointed out that radiation action on starch is considered as a more safe and environmentally friendly modification method compared to chemical methods [19]. Furthermore, high energy radiation (UV, ionizing) can be also used for modification of starch solubility or initiation of grafting process for obtaining biodegradable packaging materials [19].

## 2. Results and Discussion

### 2.1. Structure and Morphology of Native Starch

#### 2.1.1. SEM Analysis

**Figure 1:**
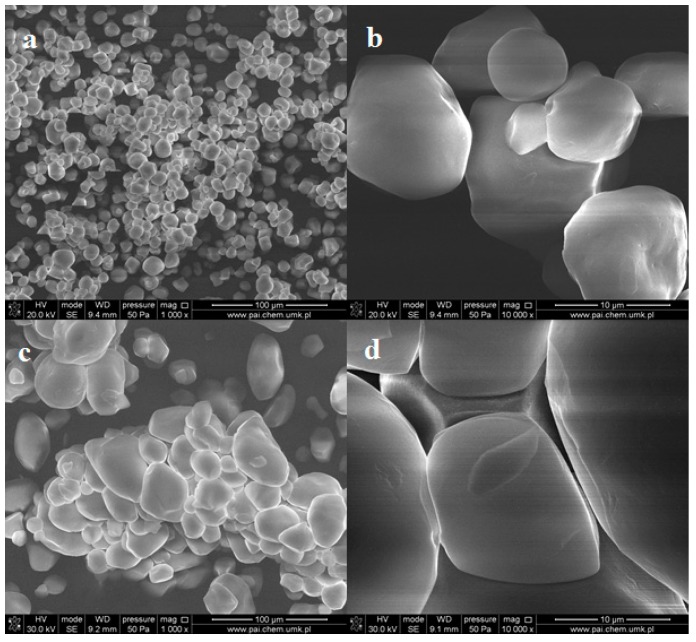
SEM images of pristine corn starch (**a**,**b**) and potato starch (**c**,**d**) in the form of powder at different magnifications.

The native starch (ST) samples in the form of powders were characterized by SEM and ATR-FTIR spectroscopy. Both types of the starch form agglomerates consisting of loosely-bound grains (Figure 1). The average size of the individual grains varies between 10 and 25 μm for corn starch (CST) and potato starch (PST), respectively.

One can notice that the shape of CST grains is close to spherical, while the PST objects take spherical and ellipsoidal shape. However, the surfaces of PST grains are smoother than that of CST. Pinholes and cavities are also observed in individual CST grains (Figure 1b).

#### 2.1.2. ATR-FTIR 

**Figure 2:**
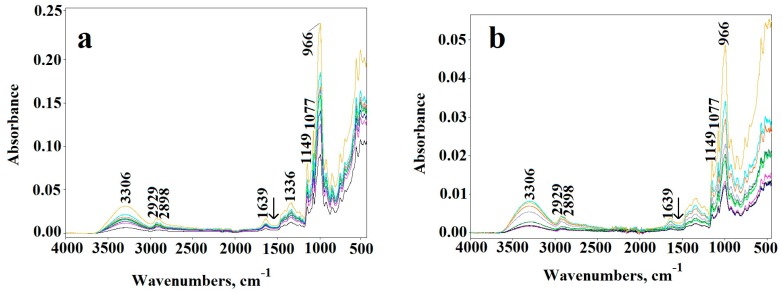
The changes in ATR-FTIR spectra of (**a**) corn starch (CST) and (**b**) potato starch (PST) after UV-treatment up to 480 min.

Infrared spectra of the unmodified corn starch do not differ from that of potato starch (Figure 2), reflecting the lack of essential differences in their chemical structure. The typical absorption bands at 3000–3600 cm^−1^ (attributed to OH stretching vibrations), 2800–3000 cm^−1^ (CH stretching), 900–1200 cm^−1^ (C–O–C stretching), 400–900, and 1200–1500 cm^−1^ (C-H, C-OH deformation) confirm characteristic polysaccharide structure [20,21].

### 2.2. Characterization of Oxidized Starch

#### 2.2.1. Determination of the Aldehyde Group Content (ALD, %)

The oxidation of starch using sodium periodate is a selective reaction which leads to the breaking of C2–C3 bond in the pyranose ring and to the creation of aldehyde groups attached to these carbon atoms (i.e., dialdehyde moieties). During oxidation of CST and PST in water dispersions containing various content of oxidizing agent—sodium periodate (SP), starch solubility was changed. Potato starch (PST) became soluble in water just after few minutes of reactions, contrary to corn starch (CST), which remained insoluble up to 1.5 h of oxidation process. After drying, the powder product was white (DAS-C1–5) or light beige (DAS-P1–5). In a strongly alkaline medium, the oxidized starch undergoes Canizzaro reaction, i.e., disproportionation of two aldehyde groups to alcohol and carboxylic groups, which allows us to determine their amount during classic acid-base titration.

The number of units containing aldehyde groups (ALD), based on alkali consumption, generally increases with rising amount of an oxidizing agent in a reaction mixture (Table 1). Although the degree of oxidation is high, the unmodified glycosidic rings are still present in the polysaccharide chains and predominate in most samples. As can be seen, the highest oxidation degree can be achieved at equal proportions of starch and periodate (1:1). Moreover, the corn starch, at the 1:1 ratio, is more susceptible to oxidation than the potato starch, which was confirmed by the highest percentage content of dialdehyde groups (67%). The differences between both starches may result from different amount of amylose in these starches. A higher content of amylose in the corn starch (28%) than in the potato starch (21%) promotes the modification process. The amylose has a linear structure and low molecular weight. Oxidation occurs mainly in the amorphous regions which are known to be more susceptible to this process [22].

Our results are consistent with the results described by Kuakpetoon and Wang [23] who found that the high-amylose starch was more susceptible to oxidation than the starch with lower amylose content.

**Figure 3:**
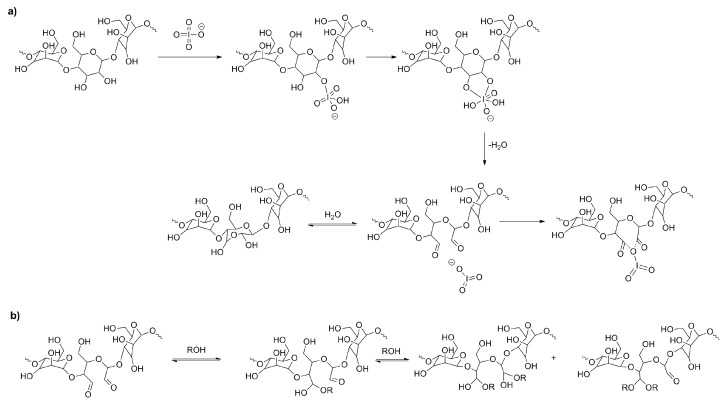
Mechanism of starch oxidation with sodium periodate (**a**), hemiacetals and acetals formation (**b**).

Moreover, dialdehyde groups, formed as a results of the ring opening and oxidation reactions, can exist in equilibrium in few forms: hemiacetals and hemialdals [24] (Figure 3). Formation of these moieties is reversible process and depends on the environment. It is necessary to remember that aldehyde groups can react with hydroxyl groups to form another starch molecule (or another unit of the same molecule). Thus, substituent denoted as R in scheme (Figure 3) is in fact a starch macromolecule. The substitution of two large fragments on the same carbon atom or on two carbons from the same unit is highly unlikely because of steric hindrances. Therefore, the structure of the hemiacetal is more probable.

The above scheme (Figure 3), showing formation of hemialdal in hydration reaction and ring closure, is based on the mechanism proposed by Guthrie [25]. 

#### 2.2.2. SEM Analysis

The morphology of the oxidized starches was different from that of the native samples, which was observed in SEM images.

**Figure 4:**
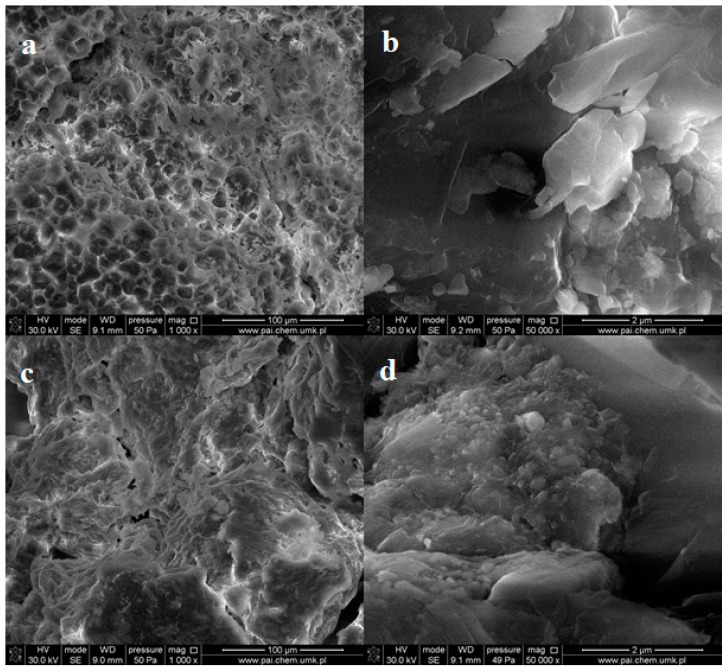
SEM images of powder dialdehyde corn starch (**a**,**b**) and dialdehyde potato starch (**c**,**d**) obtained at 1:0.5 ratio of starch to oxidant.

Generally, the globular structure was not observed after oxidation in most specimens independently from oxidant content. The globules of large diameters disappear almost completely but compact solid mass with many pores and cavities is created in these places (Figure 4a,c).

At higher magnifications, one can see a number of small spherical objects with a diameter not exceeding 0.5 μm, which are embedded in the bulk sample (Figure 4b,d). Therefore, one can state that the oxidation process leads to the grains fragmentation. This indicates easier oxidation of large starch granules.

#### 2.2.3. ATR-FTIR Analysis and ^1^H-NMR Analysis

**Figure 5:**
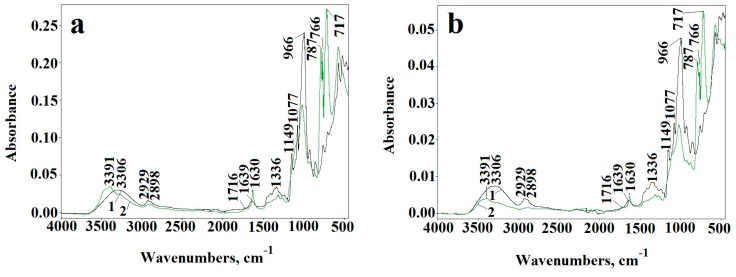
ATR-FTIR spectra of corn starch (**a**) and potato starch (**b**) (1) and after chemical oxidation to dialdehyde starch (2) and dialdehyde starch (DAS)-C3 (**a**) and DAS-P3 (**b**) starch during UV-irradiation for 480 min—arrow indicates the direction of changes after irradiation.

The ATR-FTIR spectroscopy provided the evidence of the changes in the chemical structure of dialdehyde starch (Figure 5a,b). A new carbonyl band appears at 1716 cm^−1^ in DAS-C and DAS-P spectra. Moreover, the intensity of absorption bands at 700–800 cm^−1^ as well as at 996 cm^−1^ in DAS-C and DAS-P, attributed to pyranose rings, decreases significantly. Considerable changes also occur in the vibration range of the C–O–C groups (1300–1400 cm^−1^). Maximum band at 1630 cm^−1^ is clearly shifted to 1639 cm^−1^ position, which is another proof of the dialdehyde starch formation. The maximum of hydroxyl band shifts from 3306 cm^−1^ to 3391 cm^−1^ in both types of the samples. At the same time, the width of the hydroxyl band becomes narrower in relation to band of the native starch, indicating the partial breaking of hydrogen bonds in DAS-C and DAS-P. Relatively low intensity of carbonyl band in the spectra of DAS-C and DAS-P, despite the high dialdehyde groups content, may indicate that many of these groups were transformed to hemiacetals or hemialdals. All these observations clearly confirm the oxidation reactions owing to the pyranose rings opening.

**Figure 6:**
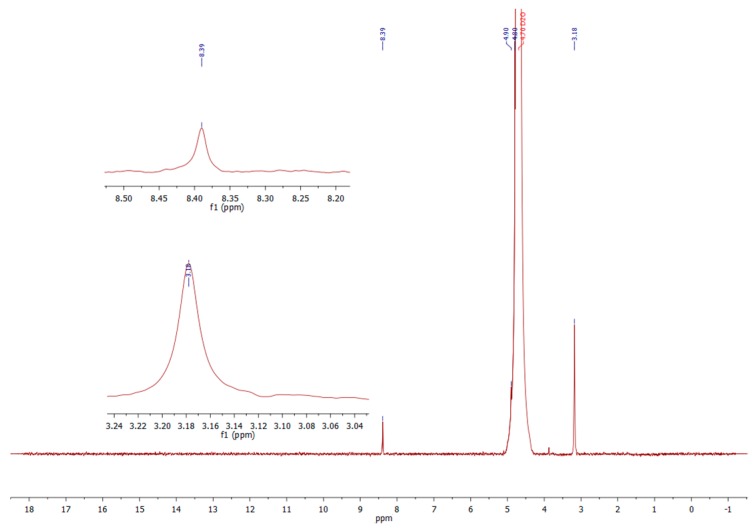
The ^1^H-NMR (D_2_O) spectrum of DAS.

^1^H-NMR spectrum of the dialdehyde starches (both types—corn and potato) showed all characteristic chemical shifts for the oxidized starch (Figure 6). Small signal at 8.39 ppm assigned to aldehyde hydrogen confirmed the successful dialdehyde starch formation. Chemical shift at 3.18 can be assigned to hydrogen atoms bonded with carbons at polymer matrix and about 5 ppm to hydrogens in hydroxyl groups (masked by D_2_O signal). This spectrum is comparable with that in literature [10].

### 2.3. Effect of UV-Irradiation

The use of polychromatic lamp emitting radiation containing also short-wavelength UV (and therefore high energy), is connected with its bactericidal action. Such radiation is used for sterilization of the materials designed for biomedical, pharmaceutical, cosmetic applications and in food industry. This method is considered to be environmentally friendly and was applied to modify polysaccharide structure in order to make crosslinked and more hydrophobic structure [26]. 

#### 2.3.1. Irradiated Native Starch

More information on the reactions induced by UV radiation in the tested starch samples were obtained on the basis of the detailed analysis of the IR spectra. 

Figure 2a,b show FTIR spectra of the unmodified starches from both sources (CST and PST) before and after irradiation. One can see a gradual decrease in all absorption bands. The most noticeable changes occur in the range of the hydroxyl bond vibrations, which is initially associated with the loss of absorbed water, and then, with the abstraction of OH groups from the macromolecules. The primary photochemical processes, due to homolytic cleavage of chemical bonds, lead to formation of free radicals, which were repeatedly detected in starch with ESR [27,28,29]. These radicals, owing to their high activity, are capable of taking part in secondary reactions, which usually result in creation of complex mixture of degradation products. 

**Figure 7:**
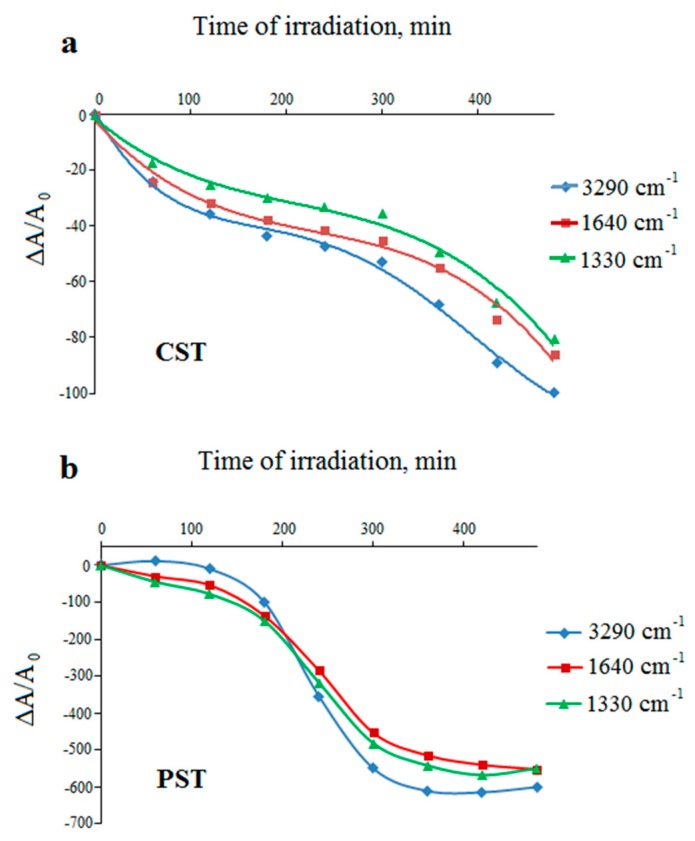
Changes in the relative absorbance of the selected bands of UV-irradiated corn (**a**) and potato (**b**) starches (unmodified) versus irradiation time.

**Figure 8:**
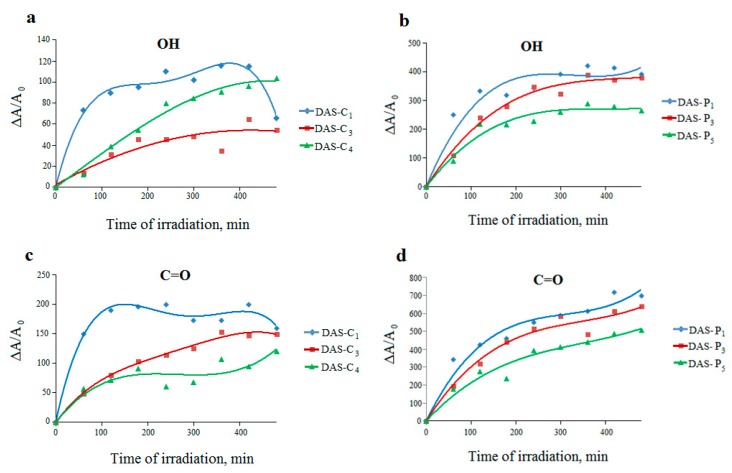
Changes in the relative absorbance of hydroxyl (**a**,**b**) and carbonyl (**c**,**d**) bands for dialdehyde corn starches (DAS-C1, DAS-C3, and DAS-C4) and dialdehyde potato starches (DAS-P1, DAS-P3, and DAS-P5) respectively, versus irradiation time.

In order to compare the behavior of the samples during UV-irradiation, absorbance values (A) of the selected bands and the relative absorbance changes were calculated. Subsequently, the relative changes, ∆A, were divided by the absorbance of the band at 2920 cm^−1^, chosen as the internal standard. This procedure allowed us to compare the results quantitatively. Figure 7 and Figure 8 show the plots of the relative absorbance changes (marked on the graphs as ∆A/A_0_) versus irradiation time for the native starches and the selected dialdehydes starches with different ALD content. 

Reduction in the intensities of all bands in both native starches (Figure 7) suggests effective photodegradation process. In the case of CST, it is relatively fast in the first hour of irradiation, but the reaction slows down between 2 and 5 h of irradiation (Figure 7a). For PST, the rapid decomposition occurs between 2 and 5 h of irradiation, but later the changes are no longer relevant (Figure 7b). Figure 7a,b show that the potato starch undergoes more efficient photodegradation than the corn starch.

#### 2.3.2. Irradiated Dialdehyde Starch

The process occurring in the dialdehyde starches under UV rays exhibits completely different trend (Figure 8). An increase in the efficiency of hydroxyl and carbonyl groups formation indicates that UV radiation causes a further oxidation of the dialdehyde starches. It can be concluded that dialdehyde starch derived from potato is still more susceptible to photooxidation than that from corn, which is confirmed by considerably greater changes in absorbance of the analyzed absorption bands in the spectra of the irradiated DAS-P samples. 

The systematically developing band at 1600–1800 cm^−1^ can be attributed to the creation of new carbonyl groups (mainly aldehydes but also probably carboxylic moieties). 

#### 2.3.3. SEM Images of Irradiated Dialdehyde Starch

**Figure 9:**
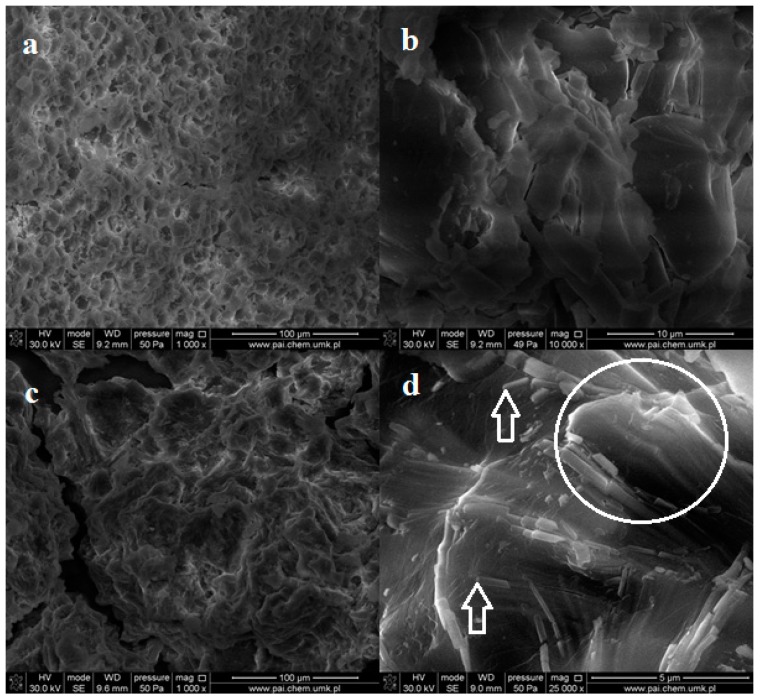
SEM images of powder dialdehyde corn starch (**a**,**b**) and dialdehyde potato starch (**c**,**d**) irradiated 8 h.

SEM images of the irradiated dialdehyde corn starch (in the powder form) do not show any changes in surface morphology (Figure 9a,b). However, the images of the exposed potato starch exhibit altered structure without grains but with numerous holes (Figure 9c). Higher magnification allows us to observe sticks and plates scattered in disordered polysaccharide bulk (Figure 9d). Some of these plates are arranged parallel, forming packages. This partially ordered structure may be a result of photodestruction of amorphous phase in the sample.

In this case, UV radiation acts as an etching agent removing outer layers and uncovering the inner ones. Such etching effect was not observed for irradiated corn dialdehyde starch, which may indicate its better photostability compared to DAS-P.

### 2.4. Surface Properties

The surface properties can affect the practical utility of the modified starch. The sample hydrophilicity was examined by the contact angle measurements using two test liquids of different polarity (polar—glycerin and apolar—diiodomethane). The measurements were used for calculating the surface free energy by the Owens–Wendt method which assumes that the surface free energy (γ_s_) is the sum of the polar (γ_p_) and the dispersive (γ_d_) component:

γ_s_ = γ_p_ + γ_d_ (1)

#### 2.4.1. Surface Properties of the Native Starch

Two native starches show remarkable different surface properties: corn starch presents lower γ_s_ and γ_p_ values, but higher γ_d_ value when compared to adequate potato starch values (Table 2). It clearly indicates more hydrophilic nature of PST, which may be related to some differences in its chemical structure (amylose/amylopectin ratio) and to different arrangement of polysaccharide chains (different content of crystalline phase).

#### 2.4.2. Surface Properties of the Irradiated Native Starch

Long-term exposure to UV rays causes a dramatic decrease in the surface free energy for both starches but unexpectedly the polar component decreases for PST, contrary to that for CST. The reduction in γ_s_ and γ_p_ values indicates the loss of the polar groups on the sample surfaces, which may be caused by both the removing adsorbed moisture and the abstraction of OH groups as a consequence of photolysis of chemical bonds. Moreover, He and co-authors postulated the surface photo-crosslinking as an explanation for the changes in contact angles of UV-irradiated potato starch [26]. Considering the changes in the solubility of the studied starch samples, it can be concluded that CST undergoes photo-crosslinking contrary to PST, which quickly becomes soluble after UV treatment.

#### 2.4.3. Surface Properties of the Dialdehyde Starch

The dialdehyde starches obtained from two botanical sources (DAS-C1,3,4 and DAS-P1,3,5) reveal slightly higher surface free energy values in relation to that of the native samples. However, much higher polar component values for DAS-C1,3,4 and DAS-P1,3,5 indicate a greater surface polarity of the dialdehyde starches with respect to the native CST and PST. This can be related to the different polarity of functional groups in starch: hydroxyl groups are characterized by smaller dipole moment (μ) than the aldehyde entities, which can be deduced from the comparison of the dipole moments of two organic substances: methanol (μ = 1.94) and acetaldehyde (μ = 2.53) [30]. Polarity of the individual chemical bonds and inter- and intramolecular interactions determine a polarity of the whole system.

It should be mentioned that the contradictory information on DAS polarity can be found in the literature. As stated in the monograph [31], transformation of OH groups into aldehyde ones made the starch surface more hydrophobic, moreover, DAS’s ability to crystallize was deteriorated.

Other publications indicated a higher hydrophilicity of DAS, if compared to the native starch [10], which has been also found in our research. 

#### 2.4.4. Surface Properties of Irradiated Dialdehyde Starch

UV-irradiation causes a decrease in surface free energies (γ_s_) and polar components (γ_p_) of the dialdehyde starches. The changes in polar components values were in the range of 17–68% and 63–74% for DAS-C1,3,4 and DAS-P1,3,5, respectively. Such behavior of these samples may be a result of the deep starch destruction related to the removal of polar functional groups from the sample surfaces. On the other hand, amylopectin, a major starch component, forms a double helix, which may be distorted during UV-irradiation. Disturbances in the helical structure can lead to conformational changes due to the rotation of C–C(OH) chemical bonds. Thus, the hydroxyl groups can be oriented towards the interior of the sample reducing the surface polarity.

Moreover, the drop in the DAS polarity may be caused by the deposition on the surface of non-polar starch contaminations (e.g., lipids) which are present in small quantities in starch specimens. It is likely that during UV-irradiation, their gradual diffusion from the inside to the outer layer may occur (an effect similar to migration of plasticizers in plastics).

Data in Table 2 can indicate no simple correlation between the carbonyl groups content in the dialdehyde starch and the surface properties.

However, spectroscopic studies indicated an increase in the amount of functional groups for both DAS-C and DAS-P during irradiation. This apparent contradiction can be explained by the difference in the thickness of the layers which are tested with both techniques. As we know, wetting test involves a top layer of thickness not exceeding 1 nm [32,33], whereas IR radiation in the ATR method penetrates the sample to approximately a few μm (micrometers) [34,35]. Thus, using both methods, we additionally obtain data about the content of polar groups placed directly on the surface and slightly below the surface, which is useful in monitoring the changes during UV irradiation of samples.

### 2.5. XRD Analysis

#### 2.5.1. XRD Analysis of the Native Starch

X-ray diffraction patterns of the native corn and potato starch (Figure 9a) indicate a partial crystallinity, which is typical of the native starches [19]. Several signals of low intensities appear at 2Θ(°): 15.2, 17.1, 17.9, and 23.0 for CST and at 17.0, 19.5, and 22.0 for PST.

The structure of starch has been explained relatively well for the recent years [2,22,36,37]. Crystalline regions are mainly composed of highly branched amylopectin while an amorphous phase is built of both the linear amylose and the amylopectin in disordered conformation. Native starch can exist in various polymorphic forms (A-type and B-type), which differ from each other in the way of double helices arrangement and in the amount of included water molecules. The third type C is a combination of A and B polymorphs. Sophisticated research methods allowed us to conclude that the granules were made up of growth rings (both amorphous and semicrystalline lamellae occur within the spherical blocklets). 

**Figure 10:**
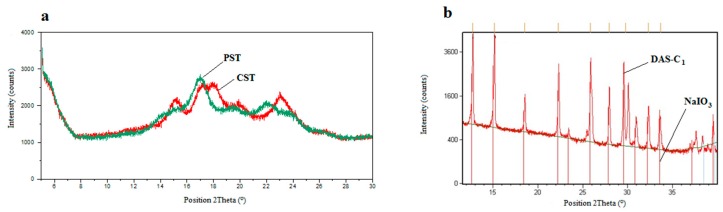
X-ray diffraction pattern of the native CST and PST (**a**), DAS-C with overlapped signals of pure NaIO_3_ hydrate (**b**).

Registered XRD pattern of CST exhibits typical A-type, whereas XRD pattern of PST shows B-type pattern (Figure 10) [37].

Both starches: CST and PST have similar crystallinity degree (about 20%). UV radiation causes a gradual disappearance of these signals with simultaneous increase in amorphous halo. It clearly indicates that the destruction occurs not only in amylose but also in more ordered fraction of amylopectin, however, the observed changes are small. It confirms that photodegradation occurs mainly in the amorphous phase. 

#### 2.5.2. XRD Analysis of the Dialdehyde Starch

XRD studies were also conducted for the dialdehyde starches. It was expected that the amorphous structure was formed as a result of the random oxidation of some glucose units. This was also suggested by other authors [6,7,38]. The lack of the oxidation on the crystallinity degree of the starch was explained by the fact that only the amorphous phase undergoes reactions [19,22]. 

Figure 10b shows XRD pattern with many narrow intensive signals indicating the presence of the crystalline product. It turned out that these signals came from the oxidizing agent reduced to iodate (V) indicating inaccurate washing of the modified starch. It should be emphasized that periodate salts are readily soluble in water. Thus, the samples were again subjected to intensive washing and shaking with distilled water and finally rinsing with acetone. The signals intensities have changed insignificantly, indicating a relatively stable combination of DAS and iodate, probably due to complexation (an example of such a structure is shown in Figure 3a). XRD pattern of the system exhibiting such strong binding may be mistakenly interpreted as a crystalline structure of the dialdehyde starch itself [39].

### 2.6. Photooxidative Degradation Mechanism of the Dialdehyde Starch

Photochemical reactions occur in systems containing chromophores absorbing the incident radiation. Although pure starch does not contain typical chromophoric moieties in its structure, but it undergoes photochemical changes due to the presence of contamination form the plant material from which it was obtained. Lipids (including phospholipids) and proteins were found in starch granules [40].

It is known that the presence of a small amount of chromophores can initiate the photochemical decomposition of polysaccharides. Quanta of UV radiation carry large enough energy to break chemical bonds of the starch. Macromolecules undergo excitation and then break down into free radicals of various types. As stated [23,27], UV-irradiation changes the structure of starch. Thus, one can observe a reduction in amylose content, in the average molecular weight and the degree of crystallinity. The mechanism of starch photodegradation has been proposed in some previous works [23,27,41,42,43]. 

**Figure 11:**
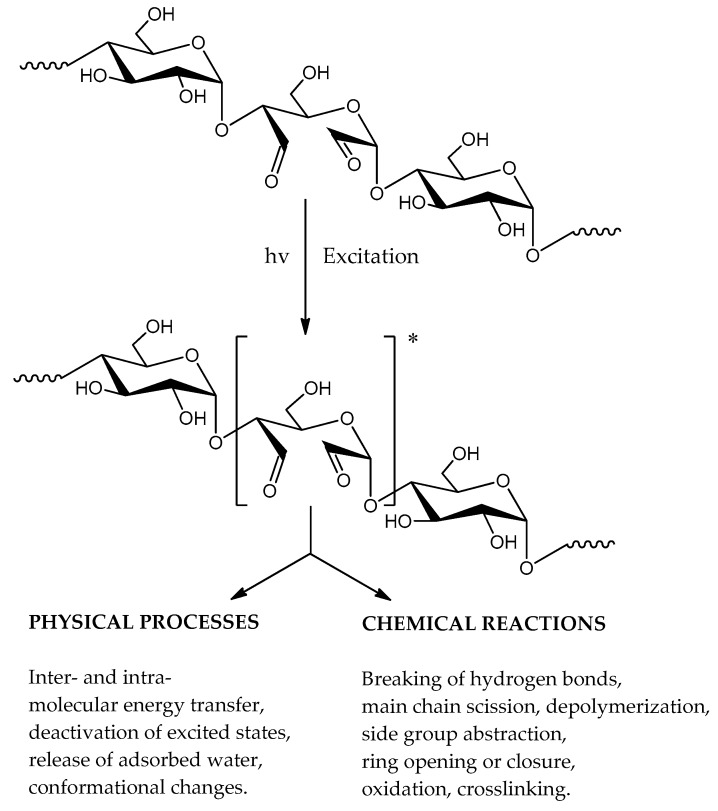
Possible photoprocesses in UV-irradiated dialdehyde starch.

The situation is different in the case of dialdehyde starch which contains a large number of carbonyl groups able to absorb UV radiation and undergo excitation (Figure 11). Thus, the aldehyde groups are the place of primary photochemical reactions in the oxidized starch. In addition to conventional free radical processes, occurring in the polymers upon UV radiation, the reactions typical of aldehydes and alcohols are expected. Norrish I type reaction involves a homolytic breaking a C–C bond in the direct vicinity of the carbonyl group (α-cleavage). Moreover, typical nucleophilic addition can take place if a proper reagent appears in the reaction medium (e.g., water or alcohols).

**Figure 12:**
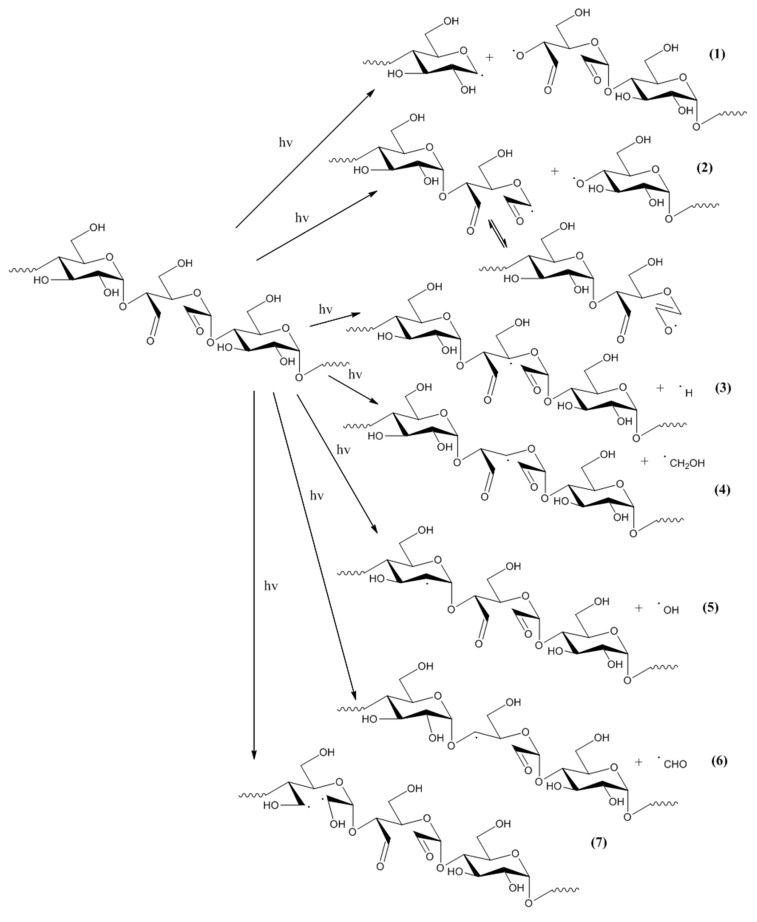
Main primary photochemical reactions in the dialdehyde starch (the hydrogen atoms in rings are omitted).

Photochemical processes in DAS can be proposed by analogy with the processes for starch. Primary photochemical reactions in DAS-C and DAS-P include the main chain scission at α-1,4 glycosidic bond, abstraction of side groups, including hydrogen atoms and opening of a glucopyranose ring (Figure 12). Free radicals, formed in the first stage, generate further reactions in the dialdehyde starch chains. This scheme does not include the reaction of the side chain abstraction and cracking of α-1,6 glycosidic bonds which are present in amylopectin. Because amylopectin dominates the amylose in both types of the native starches, its share in dialdehyde derivatives is also significant. Amylopectin, due to its ordered structure, is less susceptible to photochemical modifications than amylose but prolonged irradiation causes also the destruction of this starch component.

**Figure 13:**
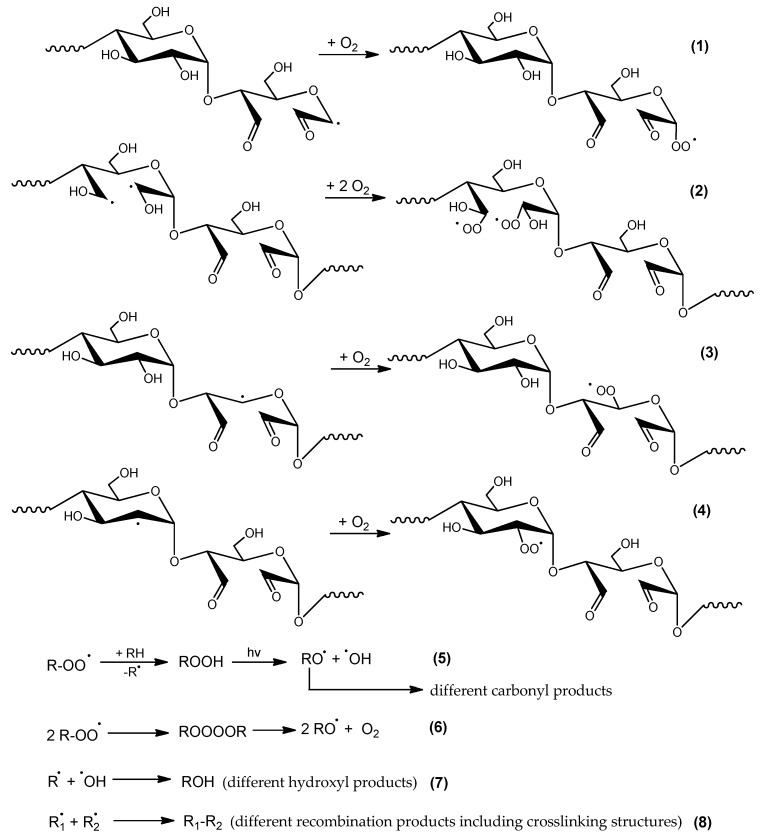
Formation of peroxyradicals in DAS-C and DAS-P and the secondary reactions (where R., R_1_ and R_2_—various radicals and/or macroradicals formed upon UV action).

Regardless of the starch component (amylose or amylopectin), small radicals such as **⋅**OH, **⋅**CHO, **⋅**CH_2_OH are formed. These radicals are characterized by high mobility and reactivity and they can abstract adjacent substituents (mostly hydrogen atoms). In these sites, macroradicals are formed, next, in air atmosphere these macroradicals react with O_2_ to form peroxy radicals (Figure 13). As shown in this scheme, the recombination of peroxy radicals leads to unstable tetraoxides that quickly decompose with the formation of molecular oxygen and alkoxy radicals.

FTIR spectra of the exposed samples indicate that the reaction of macroradicals with oxygen is dominant. Even if oxygen partially quenches the excited states of macromolecules by direct contact with them, producing oxygen in a singlet state. The singlet oxygen is an active oxidizing agent during further polymer exposure to UV.

**Figure 14:**
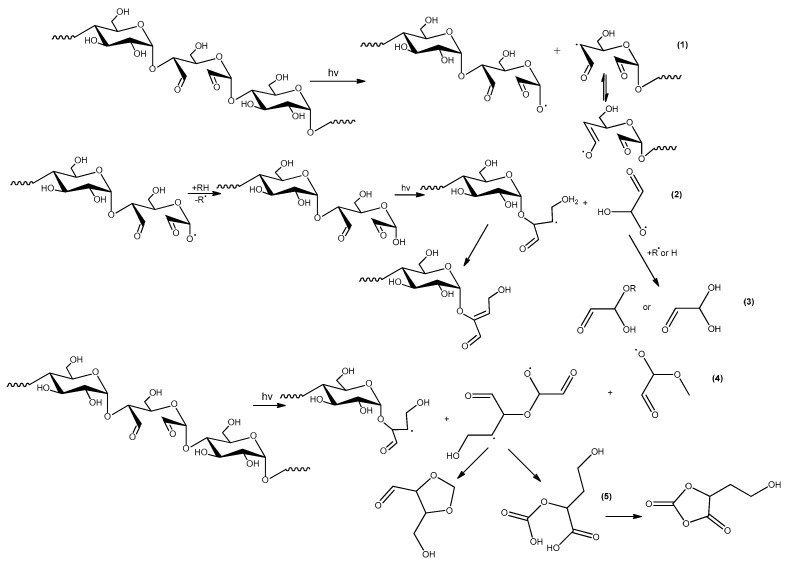
Other specific reactions in UV-irradiated the dialdehyde starch.

Another typical process, during UV-treatment of polysaccharides, is the loss of physically loosely adsorbed water as well as water more strongly bound to macromolecules (due to hydrogen bonding). The process is also observed in the case of the dialdehyde starch. Dehydration leads to the formation of double bonds in glucose units (Figure 14). Such unsaturated moieties are new chromophores in the dialdehyde starch chains, which may have an impact on the next stages of the photodegradation. The double bonds are also responsible for the absorption of UV radiation and yellowing of the irradiated samples.

Schemes in Figure 12 and Figure 13 do not include reactions in the chain units containing the dialdehyde groups in the adjacent position. The direct vicinity of these groups, which occurs in the case of the samples with high oxidation degree, implies the possibility of other specific reactions, the examples are shown in the Figure 14.

It is seen that UV radiation is responsible for the changes in the chemical structure of macromolecules and formation of low molecular weight degradation products rich in functional groups. 

The oxidation of pyranose rings during UV exposure in the presence of atmospheric oxygen can also result in the creation of the dialdehyde structures, as noted earlier [31]. In other work, it has been found that illumination of potato starch provided dialdehyde product of high water solubility and high susceptibility to enzymatic hydrolysis [44].

Nevertheless, according to our knowledge, there is no publications taking into account the effect of the oxidizer residue (reduced to NaIO_3_) on photochemical processes in DAS. NaIO_3_ has still oxidative properties and can even be reduced to free iodine, which is possible during UV-irradiation (this is confirmed by the yellow or brown color of the photooxidized products). Therefore, in the presence of the oxidizer, production of the aldehyde groups can still increase as a result of the opening of glucose rings as well as other non-selective oxidation of hydroxyl groups in C6 position. 

Due to the variety of active intermediates, secondary reactions can occur in different ways and with various efficiencies. A complex mixture of degradation products can be formed as a result of these reactions. Once initiated free-radical decomposition, it continues even if the radiation is absent. Finally, the termination process occurs when all radicals are deactivated. This happens mainly by reactions of radical recombination (Figure 13) or disproportionation. Recombination may lead to crosslinking reactions causing an increase in molecular weight, which is the opposite to the main chain scission reactions.

## 3. Materials and Methods

### 3.1. Materials

Potato and corn starch (CAS Number 9005-25-8), purchased from Sigma-Aldrich (Munich, Germany), were the raw materials for preparation of the dialdehyde starch. Both starches were white odorless powders. Sodium hydroxide and concentrated hydrochloric acid (35%) were obtained from POCh, Gliwice, Poland. Sodium periodate (SP) was purchased from Budapest (Budapest, Hungary). The glycerol and diiodomethane (pure for analysis) have been purchased from Sigma-Aldrich.

### 3.2. Dialdehyde Starch Preparation 

The dialdehyde starch (DAS) was obtained by oxidation with sodium periodate (NaIO_4_, abbreviation SP) according to the recipe in [45]. 1.5 g of potato or corn starch were dissolved in 30 mL distilled water, mixed with appropriate quantity of sodium periodate (0.7 M, 5–10 mL) and magnetic stirred at 40 °C for 3 h. When the reaction was finished, DAS was precipitated by the addition of acetone and isolated by filtration. The precipitated product was washed three times with water and acetone. Finally, DAS was dried in a room temperature for 24 h. The chemical structure of the oxidized starch has been confirmed by FTIR spectroscopy (Spectrum Two, Perkin Elmer, Waltham, MA, USA) and the content of the aldehyde groups was determined by titration analysis.

### 3.3. Determination of the Content of Aldehyde Groups (ALD, %)

The number of the aldehyde groups has been determined by acid-base titration of the modified starch solutions [45]. The content of starch units containing the dialdehyde groups was calculated from the formula:

(2) $$\mathrm{ALD},\;\%=\frac{C1V1\;-\;C2V2}{m/M}\;\times \;100\%$$

where *C*1 (mol/L) and *V*1 (dm^3^) are the concentration and volume of NaOH solution, *C*2 (mol/L) and *V*2 (dm^3^) are the concentration and volume of HCl solution, respectively; *m*—mass of the sample (g), *M*—molecular weight of the repeated unit in dialdehyde starch (*M* = 160 g/mol).

DAS (0.1 g) and NaOH solution (5 mL, 0.25 M) were added to the Erlenmeyer flask and heated in 70 °C (water bath) until the sample was dissolved. After cooling the HCl solution (7.5 mL, 0.25 M) and distilled water (15 mL) were added. Then phenolophthalein solution was added and the sample was titrated with NaOH solution (0.25 M). Procedure was triplated for each sample.

### 3.4. Preparation of DAS Films

0.2 g of DAS powder was dissolved in 20 mL of distilled water and stirred with a magnetic stirrer at 40 °C during 3 h. Then, this solution was poured onto the leveled glass plates (microscope slides or Petri dishes) to allow the solvent to evaporate. The samples are marked as follows:

CST—native corn starch
PST—native potato starch
DAS-C1–5—a sample of corn starch oxidized at different reagent ratio CST: SP
DAS-P1–5—a sample of potato starch oxidized at different reagent ratio (PST: SP), in particular, a subscript 1, 2, 3, 4, and 5 means PST:SP = 1:0.5, 1:0.7, 1:0.9, 1:1, and 1:1.1, respectively.

### 3.5. Conditions of UV-Irradiation

The dialdehyde starch and native starch films or powders were exposed to high-pressure mercury vapor lamp HPK 125 W (Philips, Eindhoven, Holland), emitting polychromatic radiation in the range of 248–578 nm. The intensity of the emitted radiation was equal to 72 W/cm^2^-UVA, 55 W/cm^2^-UVB, 18,7 W/cm^2^-UVC, and was measured with an electronic radiometer HD 9021 (Delta OHM, Caselle di Selvazzano, Italy). Maximal time of exposure was 8 h. The procedure was triplated.

### 3.6. Characterization of DAS

#### 3.6.1. Attenuated Total Reflectance Fourier Transform Infrared (ATR-FTIR)

Infrared spectra have been obtained using Spectrum Two^TM^ (Perkin Elmer, Perkin Elmer, Waltham, MA, USA) equipped with an ATR tool with diamond crystal. Spectra was recorded on over the region from 4000 to 400 cm^−1^, at a resolution 4 cm^−1^, 32 scans at room temperature.

#### 3.6.2. Scanning Electron Microscopy (SEM)

The morphology of DAS-C and DAS-P has been studied with Zeiss HamburgLEO Scanning Electron Microscope 1430 VP (Carl Zeiss, Oberkochen, Germany) without sputtering, using an adjustable vacuum mode.

#### 3.6.3. Contact Angle and Surface Free Energy

The hydrophilic/hydrophobic properties of starch were analyzed by contact angle measurements using a DSA G10 goniometer (Kruss GmbH, Hamburg, Germany). A drop of glycerin or diiodomethane was placed on the polymer film surface. The sessile drop image was recorded and digitized by camera. The drop shape analysis and determination of contact angle have been done with help of instrument software (Kruss GmbH, Hamburg, Germany). The reported contact angle value is an average at least 10 measurements for each specimen and solvent (glycerin or diiodomethane). All measurements were carried out at constant temperature (22 °C). The surface free energy was calculated by the Owens–Wendt method [46] which is one of the most commonly used calculation methods. 

#### 3.6.4. X-ray Diffraction (XRD) Measurement

X-ray diffraction pattern (XRD) were recorded with X’PertPRO diffractometer (Malvern Panalytical, Almelo, Holland) using a CuKα radiation (wavelength of 1.540 Å, nickel filtered) and High Score software. Crystallinity degree in XRD was calculated using the formula: % Crystallinity = (total area of crystalline peaks) × 100/(total area of all peaks).

## 4. Conclusions

The dialdehyde starch with different content of the dialdehyde groups was obtained by chemical oxidation of the native corn or potato starch. It was found that the corn starch was more susceptible to selective oxidation than the potato starch. During this process, the formation of the aldehyde groups on C2 and C3 carbon atoms takes place as a result of the cleavage of glycosidic rings. 

Moreover, the strong interactions between the dialdehyde starch and the residue of oxidant (IO^3−^) have been observed, which was leading to complex formation. The oxidant residue in the sample may be an additional factor accelerating the photo-oxidation. It was proved that photochemical reactions in the studied dialdehyde starches are different from that occurring in both native starches. Spectroscopic results showed a systematic increase in the amount of functional groups in DAS during UV exposure, whereas the decomposition processes were dominant for the native starches. 

Significant decrease in the surface free energy after UV-irradiation indicates efficient destruction of the sample top layers. The most profound drop in the surface polarity is not simply related to the oxidation degree of the starch. 

UV radiation also caused considerable alterations in the morphology of DAS, the granular structure disappeared in contrast to the native corn and potato starches. 

Summarizing, it can be concluded that potato starch is less resistant to UV radiation than corn starch. The dialdehyde starch obtained from PST (i.e., DAS-P) is less photostable than that obtained from CST (i.e., DAS-C), that may be caused by the differences in the structure and chemical composition of the native polysaccharides. 

The main explanation for the observed differences in the photochemical stability of the oxidized starches results from different chemical structure of the native polysaccharides. The original structure of the polysaccharides may be also partially preserved in the dialdehyde starches. A higher amount of branched amylopectin in the potato starch can be responsible for its higher resistance to UV radiation. By analogy with typical synthetic polymers (polyolefines, vinyl, or acrylate polymers), branching points are prone to UV cracking. It was reported that the polymer chains became shorter in amylose and amylopectin as a result of photoinduced depolymerisation, while amylose underwent photocrosslinking [19]. Thus, the higher amylose content in CST was the reason for slightly slower and less efficient photodegradation because crosslinks reinforced the polysaccharide structure.

Moreover, the ordered structure of A type dominates in CST, in contrast to PST in which the structure of B type was found. It seems that incompact packing of the double helices in crystallities B facilitates the penetration of oxygen, free radicals as well as active low molecular products into the interior of the helices, thus enhance their activity and simultaneously diminish starch photostability.

This study allowed us to compare the photostability of the dialdehyde starches obtained by oxidation in the same conditions. The polysaccharides studied came from two different botanical raw materials. The dialdehyde corn starch may be recommended for practical applications as more resistant to UV and characterized by a higher content of functional groups in comparison to the potato dialdehyde starch. Finally, it should be pointed out that the creation of the permanent interactions between DAS and IO_3_^−^, according to our knowledge, has not been described in the literature.

## Figures and Tables

**Table 1:** The results of acid-base titration of the oxidized starch samples obtained with different ratios of the starch to oxidant; aldehyde group content (ALD, %) is a content of units containing dialdehyde groups.

Sample	CST or PST: SP	ALD, %
DAS-C_1_	1:0.5	25
DAS-C_2_	1:0.7	29
DAS-C_3_	1:0.9	37
DAS-C_4_	1:1.0	67
DAS-C_5_	1:1.1	45
DAS-P_1_	1:0.5	21
DAS-P_2_	1:0.7	25
DAS-P_3_	1:0.9	29
DAS-P_4_	1:1.0	33
DAS-P_5_	1:1.1	33

**Table 2:** Surface free energy (γ_s_) and its dispersive (γ_d_) and polar (γ_p_) component calculated for the native and the dialdehyde starches.

Sample	Surface Free Energy [mJ/m^2^]					
	Before Irradiation			After 8 h UV-Irradiation		
	γ_s_	γ_d_	γ_p_	γ_s_	γ_d_	γ_p_
CST	34.4	28.8	5.6	19.5	6.2	13.3
PST	40.6	26.7	13.9	20.7	14.2	6.5
DAS-C_1_	37.3	20.5	16.9	32.2	27.8	4.4
DAS-C_3_	37.3	20.5	16.9	33.0	27.0	6.1
DAS-C_4_	42.6	22.9	19.7	41.1	34.8	6.3
DAS-P_1_	42.3	23.9	18.5	36.2	30.5	5.7
DAS-P_3_	42.9	21.5	21.4	28.1	12.8	15.3
DAS-P_5_	43.9	20.1	23.8	32.4	21.0	11.4
